# Prevalence and outcomes of sepsis in children admitted to public and private hospitals in Latin America: a multicenter observational study

**DOI:** 10.5935/0103-507X.20210030

**Published:** 2021

**Authors:** Daniela Carla Souza, Eliane Roseli Barreira, Huei Hsin Shieh, Andrea Maria Cordeiro Ventura, Albert Bousso, Eduardo Juan Troster, on behalf of The LAPSES Group

**Affiliations:** 1 Pediatric Intensive Care Unit, Hospital Universitário, Universidade de São Paulo -São Paulo (SP), Brazil.; 2 Pediatric Intensive Care Unit, Hospital Sírio- Libanês - São Paulo (SP), Brazil.; 3 Department of Emergency, Hospital Israelita Albert Einstein - Sao Paulo (SP), Brazil.; 4 Department of Pediatrics, Medical School, Hospital Israelita Albert Einstein - São Paulo (SP), Brazil.; 5 Hospital Municipal Vila Santa Catarina - São Paulo (SP), Brazil.; 6 Department of Pediatrics, Medical School, Universidade de São Paulo - São Paulo (SP), Brazil.; 7 Pediatric Intensive Care Unit, Hospital Israelita Albert Einstein - São Paulo (SP), Brazil.

**Keywords:** Sepsis, Septic, shock, Critical illness, Child, Hospital, public, Hospital, private, Latin America, Intensive care units, Intensive care units, pediatric, Prevalence, Mortality, Sepse, Choque séptico, Estado terminal, Criança, Hospitais públicos, Hospitais privados, América Latina, Unidades de terapia intensiva, Unidades de terapia intensiva pediátrica, Prevalência, Mortalidade

## Abstract

**Objective:**

To report the prevalence and outcomes of sepsis in children admitted to public and private hospitals.

**Methods:**

Post hoc analysis of the Latin American Pediatric Sepsis Study (LAPSES) data, a cohort study that analyzed the prevalence and outcomes of sepsis in critically ill children with sepsis on admission at 21 pediatric intensive care units in five Latin American countries.

**Results:**

Of the 464 sepsis patients, 369 (79.5%) were admitted to public hospitals and 95 (20.5%) to private hospitals. Compared to those admitted to private hospitals, sepsis patients admitted to public hospitals did not differ in age, sex, immunization status, hospital length of stay or type of admission but had higher rates of septic shock, higher Pediatric Risk of Mortality (PRISM), Pediatric Index of Mortality 2 (PIM 2), and Pediatric Logistic Organ Dysfunction (PELOD) scores, and higher rates of underlying diseases and maternal illiteracy. The proportion of patients admitted from pediatric wards and sepsis-related mortality were higher in public hospitals. Multivariate analysis did not show any correlation between mortality and the type of hospital, but mortality was associated with greater severity on pediatric intensive care unit admission in patients from public hospitals.

**Conclusion:**

In this sample of critically ill children from five countries in Latin America, the prevalence of septic shock within the first 24 hours at admission and sepsis-related mortality were higher in public hospitals than in private hospitals. Higher sepsis-related mortality in children admitted to public pediatric intensive care units was associated with greater severity on pediatric intensive care unit admission but not with the type of hospital. New studies will be necessary to elucidate the causes of the higher prevalence and mortality of pediatric sepsis in public hospitals.

## Abstract (pt)

**Objetivo:**

Relatar a prevalência e os desfechos da sepse em crianças admitidas em hospitais públicos e privados na América Latina.

**Métodos:**

Análise post-hoc dos dados do *Latin American Pediatric Sepsis Study* (LAPSES), um estudo de coorte que avaliou a prevalência e os desfechos da sepse em crianças admitidas em 21 unidades de terapia intensiva pediátricas de cinco países latino-americanos.

**Resultados:**

Dentre os 464 pacientes com sepse, 369 (79,5%) foram admitidos em hospitais públicos e 95 (20,5%) em privados. Em comparação com os admitidos em hospitais privados, os pacientes com sepse admitidos em hospitais públicos não diferiram em termos de idade, sexo, condição de imunização, tempo de permanência no hospital ou tipo de admissão, porém tiveram incidência mais alta de choque séptico, escores *Pediatric Risk of Mortality* (PRISM), *Pediatric Index of Mortality* 2 (PIM 2) e *Pediatric Logistic Organ Dysfunction* (PELOD) mais altos e taxas mais elevadas de doenças de base e analfabetismo materno. A proporção entre pacientes admitidos a partir de enfermarias pediátricas e mortalidade relacionada à sepse foi mais alta nos hospitais públicos. A análise multivariada não mostrou qualquer correlação entre mortalidade e tipo de hospital, porém, nos hospitais públicos, a mortalidade se associou com níveis mais altos de gravidade no momento da admissão à unidade de terapia intensiva.

**Conclusão:**

Nesta amostra de crianças admitidas em condições críticas em cinco países latino-americanos, a prevalência de choque séptico nas primeiras 24 horas da admissão e a mortalidade relacionada à sepse foram mais elevadas em hospitais públicos do que nos privados. A mortalidade relacionada à sepse mais elevada em crianças admitidas em unidades de terapia intensiva pediátrica de hospitais públicos se associou com maior gravidade por ocasião da admissão à unidade de terapia intensiva, porém não com o tipo de hospital. São necessários novos estudos para elucidar as causas da maior prevalência e mortalidade de sepse pediátrica em hospitais públicos.

## INTRODUCTION

Sepsis constitutes a burden on children’s health worldwide. Not only is sepsis a life-threatening condition but a substantial amount of healthcare resources are also spent treating it.^(1-5)^ Despite global efforts to improve the diagnosis and management of pediatric sepsis, such as American College of Chest Physicians/Pediatric Advanced Life Support (ACCM/PALS) guidelines for hemodynamic support of pediatric sepsis^(6)^ and the Surviving Sepsis Campaign (SSC) guidelines,^(7,8)^ sepsis-related mortality remains high, and the disease is a growing public health issue that is often neglected.^(2-4)^ This problem is even more of a concern in low-middle income countries, where low vaccine coverage rates and poor sanitary conditions lead to a high frequency of infectious diseases.^(9-11)^

Epidemiological data on pediatric sepsis in developing countries, however, are still scarce and incomplete. In 2015, World Health Organization (WHO) reported that nearly 5.9 million deaths occurred in children under 5 years of age. Most of these deaths occurred in developing countries and were related to severe infectious diseases, such as pneumonia, diarrhea, and malaria, where the term “severe” is used to describe conditions presenting with signs of poor perfusion, such as acidosis and hypotension, hallmarks of severe sepsis and septic shock.^(9)^ These data suggest that sepsis is the leading cause of death in children in developing countries.

Limited data on pediatric sepsis in Latin America have been published until now. Most information derives from studies with small sample sizes and heterogeneous populations. These studies show high pediatric sepsis-related mortality, ranging from 25% to 67%.^(12,13)^ The literature also suggests that socioeconomic features may influence the incidence and outcomes of sepsis in Latin America.^(14-16)^ Additionally, substantial inequalities in availability and access to healthcare services, as well as poor outcomes of sepsis patients admitted to public hospitals in developing countries, have been well documented.^(14,17-20)^ These aspects are especially relevant in Latin American countries, such as Brazil, where only 20% to 25% of the population has access to private healthcare insurance.^(21)^

Differences in the prevalence and outcomes of pediatric sepsis between public and private hospitals have never been investigated in Latin America. The objective of this study was to report the prevalence of sepsis within the first 24 hours at admission and sepsis-related mortality in pediatric intensive care units (ICUs) of public and private hospitals in Latin America.

## METHODS

We conducted a *post hoc* analysis of the Latin American Pediatric Sepsis Study - LAPSES^(22)^ - a prospective, multicenter, observational study conducted from June to September 2011 in 21 pediatric ICUs in five Latin American countries (Brazil, Argentina, Chile, Paraguay and Ecuador). Pediatric ICU affiliated with *Sociedad Latinoamericana de Cuidados Intensivos Pediátricos* (SLACIP) were invited to participate. In the LAPSES, all children aged 29 days to 17 years admitted to the participating pediatric ICUs during the study period were eligible for inclusion. Patients admitted to the pediatric ICU for procedures who were readmitted within 72 hours after pediatric ICU discharge and those in palliative care were excluded. The LAPSES protocol was approved by the Committees for Ethics in Research from all the participating centers. Informed Consent was obtained from the patients or legal guardians.

Clinical and demographic characteristics, laboratory data, Pediatric Index of Mortality 2 (PIM 2), Pediatric Risk of Mortality (PRISM) and Pediatric Logistic Organ Dysfunction (PELOD) scores, the origin of the patient - Emergency Department (ED), pediatric wards or other hospitals, and maternal education (as a surrogate for socioeconomic status) were registered for all the patients within the first 24 hours of pediatric ICU admission. Pediatric ICU length of stay and mortality were recorded at pediatric ICU discharge or death. Sepsis, severe sepsis, and septic shock were defined according to the International Pediatric Sepsis Consensus Conference,^(23)^ and multiple organ dysfunction syndrome (MODS) was defined as the presence of two or more organ dysfunctions according to the PELOD score. Patients were followed until pediatric ICU discharge or death. Those who were still in the pediatric ICU on the last day of the study were treated as survivors.

In this study, public hospitals were defined as hospitals for which the public health system was the main sponsor, regardless of whether they provided private care or had any partnership with private institutions. Private hospitals were defined as those whose financing was mainly provided by the patients themselves or health insurance companies, and teaching hospitals were defined as hospitals affiliated with medical schools or universities.^(24)^ Both hospital and pediatric ICU features, such as type of financing, type of hospital (pediatric, maternal-infant or nonpediatric), number of beds, physical structure, and availability of material and human resources, were also recorded.

### Statistical analysis

Categorical variables are expressed as absolute or relative frequencies and were compared using Pearson’s chi-square or Fisher’s exact tests. Continuous variables are expressed as the mean ? standard deviation (SD) or median and interquartile ranges (IQR), according to the distribution, and were compared using the Mann-Whitney or Kruskal-Wallis tests. The prevalence of sepsis was defined as the ratio of patients who had a diagnosis of sepsis within the first 24 hours of admission to the total number of patients included in the study. The results are reported as absolute numbers, percentages, and respective 95% confidence intervals (95%CI). The association of sepsis with pediatric ICU mortality was evaluated with a multiple logistic regression model that included the variables that showed significance levels ? 0.20 on univariate analysis. A two-sided p value < 0.05 was considered significant. Statistical analysis was performed using *Statistical Package for Social Sciences* (SPSS), version 20 (Chicago, IL, United States).

## RESULTS

Thirteen public and eight private hospitals, all located in urban areas, participated in the study, comprising a total of 257 pediatric ICU beds. Because of different timings in study approval by the local Committee for Ethics in Research, the data collection period differed among the participating pediatric ICUs: four months in nine pediatric ICUs, three months in eight pediatric ICUs and two months in four pediatric ICUs. The characteristics of the pediatric ICUs are shown in table 1. In general, public and private hospitals were similar regarding their physical structure and availability of equipment, material, and human resources. A greater proportion of private pediatric ICUs had full-time respiratory therapists and provided care for trauma and heart surgery patients, whereas a greater proportion of public hospitals had pediatric residents working daily.

**Table 1:** Comparisons between public and private pediatric intensive care unit: physical characteristics, equipment and human resources

Variable	Public	Private	p value
General characteristics and physical structure			
Type of hospital			0.48
General hospital	9 (69.2)	7 (87.5)	
Children's hospital	3 (23.1)	1 (12.5)	
Maternity hospital	1 (7.7)	0	
Type of ICU			0.92
Neonatal and pediatric	3 (23.1)	2 (25)	
Pediatric	9 (69.2)	5 (62.5)	
Adult and pediatric	1 (7.1)	1 (12.5)	
Specialized pediatric ICUs			> 0.99
No	12 (92.3)	8 (100)	
Oncology	1 (7.7)	0	
Number of hospital beds			0.05
< 100	0	2 (25)	
100 - 500	11 (84.6)	6 (75)	
> 500	2 (15.4)	0	
Number of pediatric ICU beds.	11.5 ± 6.0	13.8 ± 4.9	0.21
Care for trauma patients	6 (46.2)	8 (100)	0.01
Care for heart surgery patients	3 (23.1)	6 (75)	0.03
Care for neurosurgery patients	10 (76.9)	8 (100)	0.25
Care for organ transplant patients	4 (30.8)	0	0.13
Human resources			
Full-time chief medical officer	5 (38.5)	4 (50)	0.67
Full-time attending physician	13 (100)	8 (100)	-
Daily physician specialized in pediatric intensive care	12 (92.3)	5 (62.5)	0.25
Daily pediatric resident	13 (100)	4 (50)	0.01
Daily pediatric intensive care fellow	9 (69.2)	3 (37.5)	0.20
Full-time ICU nurse	13 (100)	8 (100)	-
Head nurse exclusive to the pediatric ICU	12 (92.3)	7 (87.5)	> 0.99
Attending nurse exclusive to the pediatric ICUs	12 (92.3)	7 (87.5)	> 0.99
Ratio of nurses/pediatric ICU beds up to 1:10	12 (100)	6 (75)	0.14
Ratio of nursing technicians/pediatric ICUs beds up to 1:2	9 (69.2)	4 (57.1)	0.65
Respiratory therapist exclusive to the pediatric ICU	6 (46.2)	8 (100)	0.01
Nutritionist working in the pediatric ICU	10 (76.9)	7 (100)	0.52
Pharmacist working in the pediatric ICU	6 (46.2)	4 (50)	> 0.99
Psychologist working in the pediatric ICU	10 (76.9)	6 (75)	> 0.99
Occupational therapist working in the pediatric ICU	5 (38.5)	1 (12.5)	0.33
Physical structure. equipment and services			
Heart monitor/bed = 1:1	12 (92.3)	8 (100)	> 0.99
Pulse oximeter/bed = 1:1	12 (92.3)	8 (100)	> 0.99
Mechanical ventilator/bed = 1:1	12 (92.3)	8 (100)	> 0.99
Capnography monitoring	10 (90.9)	7 (87.5)	> 0.99
Medical prescription system	6 (66.7)	6 (75)	> 0.99
HFOV	5 (38.5)	4 (50)	0.67
Noninvasive ventilator	13 (100)	8 (100)	-
Continuous blood pressure monitoring	12 (92.3)	8 (100)	> 0.99
CO_2_ monitoring	6 (46.2)	6 (75)	0.36
Continuous SvO_2_ monitoring	3 (23.1)	1 (12.5)	> 0.99
Intracranial pressure monitoring	9 (69.2)	8 (100)	0.13
Respiratory mechanics monitoring	11 (84.6)	8 (100)	0.50
Invasive hemodynamics monitoring	9 (69.2)	6 (75)	> 0.99
iNO	6 (46.2)	2 (25)	0.40
Full time clinical analysis laboratory	13 (100)	8 (100)	-
Full time blood bank	13 (100)	7 (87.5)	0.38
Full time operating room	13 (100)	8 (100)	-
Full time radiology service	13 (100)	8 (100)	-
Daily echocardiography in the pediatric ICU	9 (75)	6 (75)	> 0.99
Full-time computed tomography	13 (100)	8 (100)	-
Access to MRI	8 (61.5)	5 (62.5)	> 0.99
Peritoneal dialysis in the pediatric ICU	13 (100)	8 (100)	-
Hemodialysis in the pediatric ICU	10 (76.9)	4 (50)	0.346

ICU - intensive care unit; HFOV - high-frequency oscillatory ventilation; CO_2_ - carbon dioxide; SvO_2_ - mixed venous oxygen saturation; iNO - inhaled nitric oxide; MRI - magnetic resonance imaging. Results

**Figure 1:**
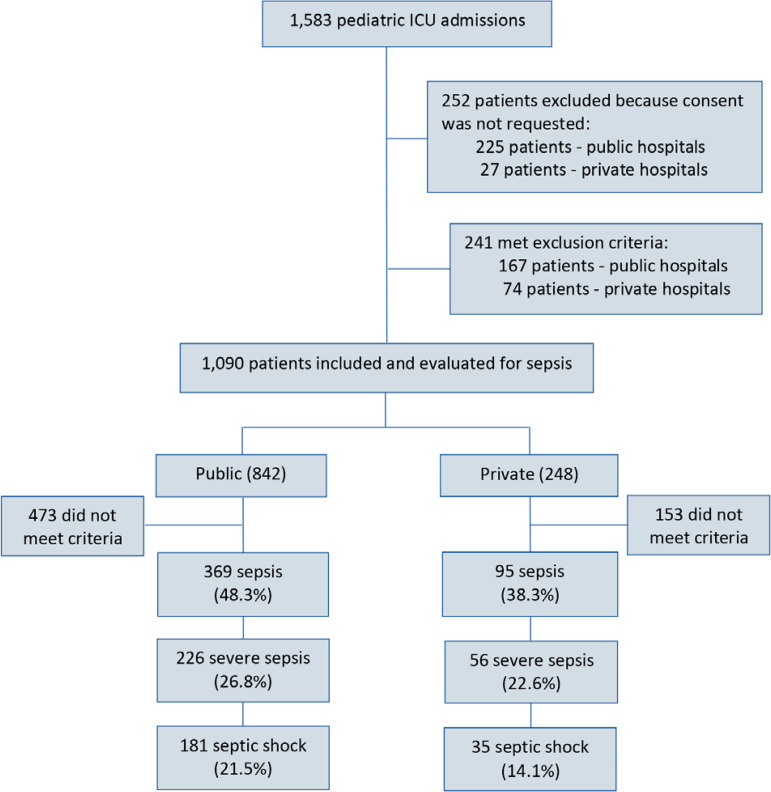
Flowchart of patients in this study. ICU - intensive care unit.

During the study period, 1,583 patients were admitted to the participating pediatric ICUs, and 1,090 were included in the study (Figure 1). The distribution of patients according to the country of origin was 599 (55%) from Brazil, 268 (24.6%) from Argentina, 129 (11.8%) from Chile, 69 (6.3%) from Paraguay, and 23 (2.3%) from Equator. Among the 464 patients who met the sepsis criteria, 369 (79.5%) were admitted to public hospitals and 95 (20.5%) to private hospitals.

The cumulative prevalence of sepsis, severe sepsis and septic shock in public *versus* private hospitals was 43.8 *versus* 38.3% (p = 0.12), 26.8 *versus* 22.6% (p = 0.17) and 21.5 *versus* 14.1% (p = 0.01), respectively.

Comparisons of clinical and demographic characteristics and of therapies administered to sepsis patients in public and private hospitals are shown in table 2. We did not observe differences in sex, age, immunization status, type of admission or pediatric ICU length of stay. Severity on admission was greater in sepsis patients admitted to public *versus* private hospitals, as shown by higher PRISM (12.7 ± 8.5 *versus* 10.1 ± 7.5, p = 0.005), PIM 2 (13.1 ± 18.6 *versus* 10.9 ± 18.3, p < 0.01) and PELOD scores (11.2 ± 9.8 *versus* 5.7 ± 7.2, p < 0.01) and the greater numbers of organ dysfunctions (2.1 ± 1.4 *versus* 1.1 ± 1, p < 0.01). Sepsis patients admitted to public hospitals also showed a higher prevalence of comorbidities (46.5% *versus* 29.8%, p = 0.003), immunodeficiency (16.2% *versus* 3.2%, p = 0.007) and maternal illiteracy (36.1% *versus* 17.9%, p < 0.01), were more frequently admitted from pediatric wards (34.5% *versus* 23.4%, p = 0.03) and had a higher frequency of mechanical ventilation (70.7% *versus* 55.3%, p < 0,01) and transfusion of blood products (43.1% *versus* 26.6%, p < 0,01) than those admitted to private hospitals.

**Table 2:** Clinical and demographic characteristics and therapeutic interventions administered to sepsis patients in public and private pediatric intensive care units

Variable	Public n = 369	Private n = 95	p value
Characteristics			
Age in months	11.3 [3.2 - 52.2]	13 [3.4 - 43]	0.77
Male	188 (51.1)	54 (57.4)	0.27
PRISM	11 [6 - 17.3]	8 [5 - 14.8]	< 0.01
PIM 2	6.3 [1.9 - 15.1]	2.5 [1 - 8]	< 0.01
PELOD	11.2 ± 9.8	5.7 ± 7.2	< 0.01
Number of organ dysfunctions (PELOD)	2.1 ± 1.4	1.1 ± 1	< 0.01
Pediatric ICU length of stay in days	7 [3 - 14]	8 [3 - 16]	0.41
Complete immunization schedule	173 (74.2)	74 (81.3)	0.17
Maternal illiteracy	104 (36.1)	15 (17.9)	< 0.01
Source of admission			0.03
ED	154 (41.8)	55 (58.5)	
Wards	127 (34.5)	22 (23.4)	
Operating room	19 (5.2)	4 (4.3)	
Other hospital	68 (18.5)	13 (13.8)	
Admission for medical reasons	335 (91)	88 (93.6)	0.83
Chronic conditions	168 (46.5)	28 (29.8)	< 0.01
Immunodeficiency	59 (16.2)	3 (3.2)	< 0.01
Organ dysfunction			
Respiratory	249 (67.8)	61 (64.2)	0.50
Cardiovascular	201 (54.8)	21 (22.1)	< 0.01
Hepatic	119 (32.6)	4 (4.3)	< 0.01
Neurologic	46 (12.5)	16 (16.8)	0.27
Hematologic	92 (25.1)	4 (4.2)	< 0.01
Renal	46 (12.5)	1 (1.1)	< 0.01
Therapies			
Mechanical ventilation	261 (70.7)	52 (55.3)	< 0.01
Vasoactive drugs	168 (45.7)	34 (35.8)	0.08
Blood products	158 (43.1)	25 (26.6)	< 0.01
Renal replacement therapy	8 (2.2)	0	0.36
Prevalence			
Sepsis	369 (43.8)	95 (38.3)	0.12
Severe sepsis	226 (26.8)	56 (22.6)	0.17
Septic shock	181 (21.5)	35 (14.1)	0.01
Mortality*	60 (16.5)	5 (5.3)	< 0.01
Sepsis	6 (1.7)	2 (2.1)	0.63
Severe sepsis	8 (2.2)	0	0.09
Septic shock	46 (12.7)	3 (3.2)	0.07
Death within 24 hours of admission	16 (4.5)	0	< 0.01
Death after 24 hours of admission	44 (12.1)	5 (5.2)	

PRISM - Pediatric Risk of Mortality; PIM 2 - Pediatric Index of Mortality 2; PELOD - Pediatric Logistic Organ Dysfunction; ICU - intensive care unit; ED - Emergency Department.

* 458 patients (public = 363; private

Comparisons between survivors and nonsurvivors are shown in table 3. Sepsis-related mortality was significantly higher in patients admitted to public *vs* private pediatric ICUs (16.5% *versus* 5.3%; p = 0.005) as was the proportion of deaths within the first 24 hours of admission (4.5% *versus* 0; p < 0.01). In public hospitals, one in four deaths from sepsis occurred during the first 24 hours after pediatric ICU admission, while in private units, no deaths were observed during the first 24 hours. We did not observe any significant difference between public and private pediatric ICUs in the mortality of patients stratified by severity (Table 2).

**Table 3:** Comparisons between survivors and nonsurvivors (global analysis)

Variable	Nonsurvivors (n = 65)	Survivors (n = 393)	Univariate analysis (p value)	Multivariate analysis p value; OR (95%CI)
Characteristics				
Age in months	16.6 [3.9 - 85.1]	11.5 [3.2 - 45.7]	0.398	-
Female	39 (60)	178 (45.5)	0.03	NS
PRISM	17 [12.8 - 28]	9 [5 - 16]	<0.001	0.005; 1.06 (1.02 - 1.11)
PIM 2	13.8 [6.4 - 41.5]	4.8 [1.4 - 11.3]	<0.001	
PELOD	21 [11 - 30]	10 [1 - 12]	< 0.001	0.001; 1.06 (1.02 - 1.11)
Number of organ dysfunctions	3 ± 1.4	1.7 ± 1.3	< 0.001	NA*
Pediatric ICU length of stay in days	8.3 ± 11.9	11.9 ± 15.4	< 0.001	NA*
Complete immunization schedule	30 (83.3)	212 (75.2)	0.28	-
Maternal illiteracy	23 (49.9)	94 (29.7)	0.077	NA*
Source of admission			0.009	
ED	23 (35.4)	184 (47.1)		1.00
Wards	33 (50.8)	114 (29.2)		0.015; 2.44 (1.19 - 5.01)
Operating room	2 (3.1)	21 (5.4)		0.506; 1.80 (0.32 - 10.24)
Other hospital	7 (10.8)	72 (18.4)		0.486; 1.42 (0.53 - 3.820
Admission for medical conditions	63 (96.9)	355 (90.8)	0.311	-
No. of chronic conditions			< 0.001	0.003
< 2	40 (61.5)	321 (81.9)		1.00
≥ 2	25 (38.5)	71 (18.1)		2.74 (1.4 - 5.36)
Organ dysfunction				
Respiratory	55 (85.9)	250 (63.8)	< 0.001	NA*
Cardiovascular	43 (67.2)	177 (45.2)	< 0.001	NA*
Hepatic	34 (53.1)	88 (22.6)	< 0.001	NA*
Neurologic	14 (21.9)	48 (12.2)	0.037	NA*
Hematologic	25 (39.1)	71 (18.1)	< 0.001	NA*
Renal	18 (28.1)	29 (7.4)	< 0.001	NA*
Type of hospital			0.005	NS
Public	60 (92.3)	303 (77.1)		
Private	5 (7.7)	90 (22.9)		

OR - odds ratio; 95%CI - 95% confidence interval; NS - nonsignificant; PRISM - Pediatric Risk of Mortality; PIM 2 - Pediatric Index of Mortality 2; PELOD - Pediatric Logistic Organ Dysfunction; ICU - intensive care unit; NA - not applicable; ED - Emergency Department. The association of sepsis with pediatric ICU mortality was evaluated with a multiple logistic regression model that included the variables that showed significance levels ≤ 0.20 on univariate analysis.

* A significant variable in the univariate analysis that was not included in the multivariate analysis. Results expressed as median [interquartile range], n (%) mean ± standard deviation.

After multivariate analysis, PRISM (odds ratio - OR = 1.06, 95%CI 1.02 - 1.11, p = 0.005) and PELOD scores (OR = 1.06, 95%CI 1.02 - 1.11, p = 0.001), the presence of two or more comorbidities (OR = 2.74, 95%CI 1.40 - 5.36, p = 0.001) and admission from pediatric wards (OR = 2.44, 95%CI 1.19 - 5.01, p = 0.015) remained associated with mortality in sepsis patients, but a significant association between the type of hospital (public or private) and mortality was no longer observed (OR = 1.46, 95%CI 0.52 - 4.09, p = 0.477).

## DISCUSSION

In this sample of critically ill children from five countries in Latin America, the prevalence of septic shock within the first 24 hours at pediatric ICU admission was significantly higher in public hospitals than in private hospitals. Our study shows that despite no significant differences observed between public and private pediatric ICUs regarding the physical characteristics and the availability of material and human resources, sepsis-related mortality was higher in children admitted to public hospitals. This difference in mortality was associated with greater disease severity on admission, as shown by the higher severity and organ dysfunction scores and the greater proportion of patients with prior comorbidities and septic shock on admission.

Our study did not elucidate the cause of the higher prevalence of septic shock within the first 24 hours of pediatric ICU admission and higher sepsis-related mortality in children admitted to public hospitals. These differences may be related to reduced levels of immunization, lower maternal illiteracy, increased numbers of comorbidities and greater disease severity. Other authors have suggested that disease severity observed in patients with sepsis admitted to the pediatric ICU may be related to delays in diagnosis and treatment and late admission to the hospital and ICU, leading to higher mortality. In a study that analyzed factors associated with sepsis mortality in adults admitted to private and public hospitals in Brazil, Conde et al. showed that admission to public hospitals was related not only to higher mortality but also to the delayed recognition of sepsis and a greater number of organ dysfunctions on ICU admission.^(25)^ In a well-designed trial, Machado et al.^(26)^ did not observe a difference in sepsis-related mortality between patients in private and public hospitals, which may suggest that better outcomes in patients with sepsis are not simply related to health insurance payments but to healthcare system characteristics, education on sepsis and implementation of quality improvement programs that can be successfully implemented in both public and private health-care services.^(27,28)^

In another study that evaluated the epidemiology of pediatric sepsis in Colombia, Jaramillo-Bustamante et al. reported that approximately 50% of the patients were admitted at a late stage of septic shock, and more than 40% had MODS on admission, which resulted in high mortality (34%) among those children.^(16)^ The authors suggested that late pediatric ICU admission was related to greater morbidity and mortality and resulted in high social and economic costs. Additionally, they observed that patients with low socioeconomic status had a higher probability of getting sick than did wealthier patients: 75% of septic children were classified in a low socioeconomic stratum, which was unrelated to their access to private pediatric ICUs. The association of mortality with illness severity has also been reported by Odetola et al., who showed that higher severity scores and increased numbers of comorbidities and organ dysfunctions were associated with higher mortality and longer pediatric ICU length of stay, while the type of hospital (children’s or nonchildren’s hospital) was not.^(29)^ Similarly, in our study, sepsis-related mortality correlated with the patients’ characteristics, such as PRISM and PELOD scores, the presence of two or more comorbidities and admission from wards, but not with the type of hospital (public or private).

Previous studies have identified possible barriers to the early diagnosis and treatment of children with severe diseases, which may explain the poor condition of the patients admitted to public hospitals in our study.^(30,31)^ The first barrier relates to the lack of knowledge about the problem among the public, as well as the low socioeconomic status of the population admitted to public hospitals.^(15,30,32,33)^ Whereas minimal awareness of sepsis has been documented in Europe and in the United States, poor socioeconomic indicators such as low income, illiteracy, and poor maternal schooling have been associated with childhood mortality in developing countries.^(15,29,32)^ In our study, sepsis patients admitted to public hospitals showed a higher prevalence of maternal illiteracy, which may have contributed to delayed diagnosis and treatment. Gavidia et al., in El Salvador, observed an association between maternal illiteracy (OR 3.06, 95%CI 1.09 - 8.63, p = 0.034) and sepsis in children undergoing cancer treatment.^(15)^ In that country, more infectious and sepsis-related deaths occurred in those with longer travel times to the hospital (OR 1.36, 95%CI 1.03 - 1.81, p = 0.031) and in families with an annual household income < US$ 2,000 (OR 13.90, 95%CI 1.62 - 119.10, p = 0.016). These authors suggest that low socioeconomic status (maternal illiteracy, longer travel times and poverty) is associated with delays in the diagnosis of infections and sepsis and in the treatment of critically ill children, and, consequently, with the prognosis of these patients.

Other identified difficulties could be related to poor and heterogeneous access to healthcare services, a shortage of pediatric ICU beds, lack of specialized services for the transport of critically ill children and late referrals to the pediatric ICU.^(18,19,34,35)^ Another obstacle is associated with limited training of emergency and primary care pediatricians in the early recognition and management of sepsis as well as with low adherence to the ACCM/PALS pediatric sepsis guidelines.^(36-38)^ Finally, the degree of health professional specialization among providers who care for critically ill children may also influence the outcomes.^(38,39)^

In our study, 25% of sepsis-related mortality in public hospitals occurred during the first 24 hours after pediatric ICU admission, while in private units, no death was observed during the first 24 hours. Similar findings have been previously reported. Recently, Weiss et al., in a retrospective observational study at two academic children’s hospitals (ER and pediatric ICU) in the United States, observed that 25% of sepsis-related mortality occurred within 1 day of severe sepsis recognition and 35% occurred within 3 days.^(40)^ Contrary to the author’s hypothesis, refractory shock leading to early death of sepsis recognition is not rare in severe pediatric sepsis. These early deaths, in general, were related to delays in diagnosis and treatment and poor adherence to guidelines for hemodynamic support of pediatric sepsis.

Another point that arose is the fact that despite the greater severity of patients admitted to public pediatric ICUs, we found no difference in pediatric ICU length of stay between public and private hospitals. This finding may be due to earlier deaths (in the first 24 hours of pediatric ICU admission) in public hospitals or later discharge in private pediatric ICUs, where demand for beds is generally lower. However, the study design did not allow us to assess the causes of similar pediatric ICU lengths of stay between public and private hospitals.

The poor adherence to the published sepsis recommendations may be related to the nonuniform applicability of the guidelines to different health service settings (i.e., ED or pediatric ICU, public or private hospitals, developed or developing countries). It has been suggested that to assure greater adherence and to reduce sepsis-related mortality, sepsis treatment guidelines should be adjusted to different situations, which has been recently included in the new Surviving Sepsis Campaign International Guidelines for the Management of Septic Shock and Sepsis-associated Organ Dysfunction in Children.^(8)^ Greater emphasis on educational efforts to improve physician skills in the early recognition and management of pediatric sepsis may also improve the outcomes.

This is, to the best of our knowledge, the first study to compare the prevalence and outcomes of pediatric sepsis between public and private pediatric ICUs in Latin America. The multicenter and international nature of this study allowed data analysis of patients from several geographic areas and different socioeconomic statuses, contributing new knowledge about pediatric sepsis scenarios in this subcontinent. This finding indicates an opportunity for improvement in the care of septic children in this setting. Some limitations of this study, however, should be acknowledged. First, we included pediatric ICUs from five countries, all of which were located in urban areas, and the sample was not randomized; thus, our results may not broadly represent the spectrum of pediatric sepsis in Latin America. Second, the authors reported pediatric ICU mortality but not hospital mortality. However, it should be noted that the number of deaths verified in our pediatric ICU is certainly related to the episode of sepsis itself, while late mortality, after discharge from the pediatric ICU, may have been due to other events that were not related to sepsis. Third, nearly one-third of the patients admitted to the pediatric ICU were not included in the study, most of them because of admissions on weekends, when the researchers were not present. Fourth, although we did not observe differences in physical structure or human resources, differences in qualitative aspects between private and public pediatric ICUs, such as continuing education of the health care staff, equipment quality and the presence of quality control protocols, may not be ruled out. Fifth, despite the data collection and publication interval (data were collected in 2011 and this study is being published in 2021), the coverage rate for private healthcare insurance from 2011 to 2021 has not changed significantly, with a drop in the growth in the number of beneficiaries who have access to private health insurance.^(21)^ Additionally, in recent years, there have been few changes in guidelines for pediatric sepsis and infrastructure in Latin America. Finally, we did not evaluate the impact of therapeutic interventions or the time elapsed between the onset of sepsis symptoms and the initiation of resuscitation and pediatric ICU admission, which are well-known factors that influence sepsis outcomes.

Decreasing sepsis-related mortality in childhood is still a challenge worldwide. Sepsis-related issues are even more alarming in developing countries, where infectious diseases are more prevalent and economic resources are limited. Implementation of simple solutions to improve the diagnosis and management of sepsis, such as educational programs for the public, training of healthcare providers, early fluid resuscitation, antibiotic administration and referral to the pediatric ICU, are cost-effective measures that may favorably impact sepsis-associated mortality in ICUs in Latin America.^(41,42)^ Efforts to optimize the early recognition and prompt management of pediatric sepsis prior to pediatric ICU admission may improve the prognosis of children receiving medical attention in public hospitals in these countries.

Future studies, with specifically designed trials, may help to elucidate the causes of the higher prevalence and mortality of pediatric sepsis in public hospitals in Latin America and the impact of social and economic factors, the distribution and access to healthcare resources, educational programs and quality of care on the outcomes of pediatric sepsis in Latin America. New studies should also assess the impact of adherence to guidelines for hemodynamic support of pediatric sepsis, the delay between diagnosis and treatment and pediatric ICU and hospital admission, the role of community-acquired sepsis and healthcare-related infections, the role of chronic diseases, the role of immunization schedule and nutritional status on sepsis prevalence and mortality, and the long-term mortality and morbidity of sepsis patients in this region.

## CONCLUSION

In this sample of critically ill children from five countries in Latin America, the prevalence of septic shock within the first 24 hours at admission and sepsis-related mortality were higher in public hospitals than in private hospitals. Higher sepsis-related mortality in children admitted to public pediatric intensive care units was associated with greater disease severity on admission to pediatric intensive care units. Differences in material and human resources between public and private hospitals did not influence mortality in the patients in our study.
